# Flight Characteristics of *Bactrocera dorsalis* Associated with Long-Distance Migration

**DOI:** 10.3390/insects17020203

**Published:** 2026-02-14

**Authors:** Naoya Hidaka, Kohei Nishiya, Yudai Masuoka, Akiya Jouraku, Yong-Jun Yang, Chia-Jung Ho, Yu-Bing Huang, Akira Otuka

**Affiliations:** 1 Institute for Plant Protection, National Agriculture and Food Research Organization, Koshi 861-1192, Kumamoto, Japannishiya.kohei662@naro.go.jp (K.N.); 2 Institute for Plant Protection, National Agriculture and Food Research Organization, Tsukuba 305-8605, Ibaraki, Japan; masuoka.yudai003@naro.go.jp; 3 Institute of Agrobiological Sciences, National Agriculture and Food Research Organization, Tsukuba 305-8634, Ibaraki, Japan; jouraku.akiya941@naro.go.jp; 4 Applied Zoology Division, Taiwan Agricultural Research Institute (TARI), Wufeng District, Taichung 413008, Taiwan

**Keywords:** oriental fruit fly, takeoff time, low temperature response, flight duration, flight mill

## Abstract

Recently, individuals of the oriental fruit fly and its relatives (*Bactrocera dorsalis* complex)—previously eradicated in Japan—have occasionally been captured in surveillance traps in the Kyushu District of western Japan, suggesting possible overseas migration. However, as this species generally is not considered capable of long-distance flight, its potential for overseas migration remains largely unexplored. Moreover, this emerging situation in East Asia poses an increasing risk to agricultural industries, highlighting the need to develop a migration prediction model to provide early warnings of their arrival, for which understanding the species’ flight characteristics is essential. In this study, flight experiments were conducted using young first-generation adults that emerged from larvae collected in Taiwan. Outdoor observations revealed that *B. dorsalis* actively initiates flight around 10:00 and sunset. Flight mill experiments under varying temperature conditions indicated that flight activity ceased at temperatures between 16.2 and 16.5 °C. Furthermore, a 24-h flight test demonstrated that some individuals were capable of sustaining flight for over 7 h, suggesting a potential capacity for long-distance migration. These findings contribute to the development of a migration flight model and enhance our understanding of the flight behavior associated with long-distance migration in *B. dorsalis*.

## Simple Summary

Recently, individuals of the oriental fruit fly and its relatives (*Bactrocera dorsalis* com-plex)—previously eradicated in Japan—have occasionally been captured in surveillance traps in the Kyushu District of western Japan, suggesting possible overseas migration. Generally, however, this species is not considered capable of long-distance flight. This emerging situation in East Asia highlights the need to understand the species’ flight characteristics associated with long-distance migration, which is essential for developing a migration prediction model to provide early warnings of their arrival and ensure effective pest control. In this study, flight experiments were conducted using young first-generation adults originating from Taiwan. Outdoor observations revealed that *B. dorsalis* actively initiates flight around 10.00 and sunset. Flight mill experiments under varying temperature conditions indicated that flight activity ceased at temperatures between 16.2 and 16.5 °C. Furthermore, a 24-h flight test demonstrated that some individuals could sustain flight for over 7 h, suggesting a potential capacity for long-distance migration. These findings improve our understanding of *B. dorsalis* flight behavior and provide a basis for migration model development.

## 1. Introduction

The oriental fruit fly and its relatives, comprising the *Bactrocera dorsalis* (Hendel) complex [1] (Diptera: Tephritidae), are major agricultural pests native to tropical and subtropical Asia. The larvae are highly polyphagous, infesting a wide range of fresh fruits and vegetables from various host plants, including citrus, carambola, guava, mango, papaya, peach, and pear [1,2,3]. In regions where this species is present or has established, the export and domestic movement of fruits and vegetables are often restricted, resulting in substantial economic losses for the countries that rely on these products’ exports to boost their economy [4].

In Japan, the oriental fruit fly was previously found in the Southwestern Islands (Ryukyu Islands) located on the periphery of the East China Sea (see Figure S1 for location), and on the Ogasawara Islands in the Pacific Ocean. It was successfully eradicated in 1986 through a male annihilation technique using methyl eugenol as an attractant—a process that spanned 18 years and cost approximately 5 billion JPY [5]. However, in the years following eradication, individuals presumed to have migrated from Taiwan Island, or the Philippines were intermittently captured in surveillance traps in the Southwestern Islands of Okinawa and Kagoshima Prefectures [6,7,8]. These occurrences are believed to be driven by the species’ strong flight capacity and meteorological factors such as monsoons [7]. This interpretation is supported by the following quarantine regulation and observations:

In Japan, the import of host plants for *B. dorsalis* is generally prohibited, and stringent plant quarantine measures are enforced by the Ministry of Agriculture, Forestry and Fisheries at seaports and airports. While individual flies suspected of being “hitchhikers” on travelers or imported goods are occasionally discovered near these ports, such instances are typically sporadic and involve only isolated individuals. This suggests that these cases arise from the incidental transport of a very small number of individuals rather than mass emergence from illegally imported host material.

In contrast, individuals captured in recent years in the Southwestern Islands and along the coastal areas of Kyushu were located far from international trade ports. Furthermore, multiple individuals were frequently trapped simultaneously across several locations. Just prior to these captures, strong atmospheric currents associated with the passage of depressions or fronts were often observed [6,7,8]. These circumstances indicate that the recent incursions of the oriental fruit fly into Japan are primarily driven by the species’ own flight capacity facilitated by favorable meteorological conditions, rather than through human-mediated pathways. Consequently, these capture events are best characterized as long-distance migration.

Historically, captures of oriental fruit flies in surveillance traps within the Kyushu District—located north to the Southwestern Islands and one of the Japanese main islands (Figure S1)—were rare and mostly limited to isolated cases involving hitchhiking with cargo near ports and airports. However, in 2020, a large number of individuals were trapped in Kagoshima Prefecture and surrounding southern islands (Figure S1) [9]. In the following year (2021), similar captures occurred along the western coastal areas of Nagasaki, Kumamoto, and Kagoshima Prefectures [10]. In 2022, both trap captures, and transient larval infestations were reported on Yakushima and Koshikijima Islands, located in southern Kagoshima Prefecture [10]. These findings raised concerns about an increasing risk of migration to, and potential establishment in, the Kyushu District. Note that the eradicated status in Japan has been sustained until now through the intensive initial control measures, including the application of wooden boards soaked with methyl eugenol and insecticide, removal of host plants around trapping areas, and subsequent confirmation of zero captures. These measures have been consistently implemented in all cases on all major inhabited islands.

The recent migration patterns of this species likely reflect changes in climatic and environmental conditions, necessitating preparedness for emerging risks. To enable early detection of immigrants and effective control of both incoming individuals and their offspring, it is crucial to develop a comprehensive migration analysis framework and predictive simulation tool covering the entire Kyushu region. Enhancing the accuracy of such methods requires the accumulation of empirical knowledge on flight characteristics, particularly from studies conducted in the species’ native range using freshly emerged individuals.

Insect flight behavior is influenced by a complex array of environmental factors, including temperature, humidity, wind direction, and wind speed, all of which fluctuate dynamically throughout the day. In backward trajectory analysis used to trace insect movement paths, the timing of the departure point at the source can significantly affect the results. Therefore, understanding the diurnal flight activity and peak flight times under field conditions is critical. Most previous studies have relied on male-attractant traps using methyl eugenol [11,12,13], which may reflect the rhythm of chemical responsiveness rather than actual flight activity, and do not account for female flight behavior.

Atmospheric temperature decreases with altitude, and the maximum flight altitude of insects is constrained by their thermal tolerance. Because wind direction often varies with altitude, the temperature threshold at which flight ceases can significantly influence predicted flight trajectories and destination areas in trajectory analysis. Although flight temperature thresholds for this species have been reported [14,15], these studies were based on laboratory-reared populations from different regions, which may exhibit altered flight characteristics due to generational rearing effects. In related species such as the melon fly (*Zeugodacus cucurbitae* (Coquillett) [= *Bactrocera cucurbitae*]) and the solanum fruit fly (*Bactrocera latifrons* (Hendel)), prolonged laboratory rearing has been shown to reduce flight capability [16,17]. Therefore, accurate assessment of flight capability requires measurements using wild or near-wild generations.

Furthermore, long-distance migration across oceanic barriers from the native range in tropical and subtropical Asia to Japan necessitates sustained flight capability in migratory insects. However, previous studies have reported limited flight durations (<3.6 h) or distances (<50 km) for this species [18,19,20,21], and it is generally not considered capable of long-distance flight spanning hundreds of kilometers. Consequently, its maximum flight duration in relation to overseas migration remains largely unexplored.

To address these gaps, we conducted flight experiments in Taiwan—a region considered as a part of the species’ native range and one of possible migration sources to Japan [7,8]—using first-generation adults that emerged from naturally infested fruits. The objectives were to determine the peak time of flight activity and the temperature threshold for flight cessation. Based on these findings, we further investigated the maximum flight duration under an optimal temperature condition. These data are essential for improving the accuracy of backward trajectory analysis and a predictive simulation model used in migration risk assessments of the oriental fruit fly.

## 2. Materials and Methods

### 2.1. Tested Insects

Fruits infested with larvae of *B. dorsalis* (guava, *Psidium guajava* L.; pomelo, *Citrus maxima* (Burm.) Merr. (1917); Citrus sp.; peach, *Prunus persica* (L.) Batsch (1801)) were collected in orchards in Taichung, Hualien, and Tainan Cities, Taiwan. Details of the collection sites and dates are provided in Table S1 in the Supplementary Information. The infested fruits were stored in a rearing room of the Taiwan Agricultural Research Institute (TARI, Taichung City, Taiwan) under controlled conditions (25.0 ± 2.0 °C, 70 ± 10% RH, 12L:12D photoperiod, light on from 6:00 to 18:00 local time) until adult emergence. Emerged adults were fed a mixture of sugar and protein hydrolysate (United States Biochemical Co., Salem, MA, USA), along with agar-based water or sugar water, and subsequently used for flight experiments. Adult *B. dorsalis* specimens used in the experiments were identified based on morphological characteristics. The specimens emerged from different species of fruits. While this may have some influence on the insects’ flight performance, we consider it to be beyond the scope of this study, as the adult diet remained consistent across all groups.

### 2.2. Outdoor Takeoff Experiment

To determine the peak flight activity time of adult *B. dorsalis* under field conditions, a takeoff experiment was conducted using a vertical cage placed outdoor on the campus of the Taiwan Agricultural Research Institute (24.03° N, 120.69° E). During the study period, sunrise and sunset occurred at approximately 06:00 and 17:20, respectively. The cage was constructed from metal hanger racks (SeiwaPro Co., Osaka, Japan) and 1-mm mesh nettings (Nihon Widecloth Co., Osaka, Japan), measuring 139.7 cm in height and 25.8 cm square at the base (Figure S2a). The cage used in this study featured a unique size and shape with no reference. The specific heights and base area were chosen to facilitate the observation of entire takeoff sequences. It was placed in a sunlit wind-sheltered area between two laboratory buildings on the TARI campus. Adults were reared as described in the previous section and used at 8–16 days post-eclosion, as newly emerged individuals exhibit reduced flight ability [15,21]. Specific details of the tested insects are listed in Table S1.

Ten adults were placed in a small wooden box (release box in Figure S2b), excluding individuals showing abnormal behavior or impaired wing movement. Ten minutes prior to the experiment, the box was placed at the bottom of the cage for acclimation. To prevent overheating, direct sunlight was blocked until the experiment began. At the start, the box was opened by pulling a string attached to a transparent acrylic sliding top cover, simultaneously releasing the flies. The number of individuals reaching the top section of the cage (above 114.7 cm) within 5 min was recorded, along with the individual times taken to reach the top.

This procedure was repeated at each time interval (6:00, 8:00, 10:00, 12:00, 14:00, 16:00, and sunset ~17:20 local time), using 10 males and 10 females per time slot, with three replicates. *Bactrocera dorsalis* is inherently diurnal and crepuscular, with no confirmed activity during the night. Based on these behavioral traits and the practical limitations of nighttime observation, we adopted the current experimental design without nighttime test. Temperature and humidity were recorded using a thermohydrometers (TR72A2, T&D Co., Matsumoto, Japan), and wind speed was measured using an anemometer (testo 405i, Testo Co., Yokohama, Japan).

### 2.3. Low-Temperature Response Test

To investigate the flight cessation temperature of adult *B. dorsalis*, a flight mill apparatus (Figures S3 and S4) was installed in a temperature-controlled incubating room (24L:0D). The rotor had an arm length (rotation radius) of 60 mm and a weight of 93.7 mg (Figure S3); the rotor needle was 40 mm long and was supported by two Phillips head (+) screws. An electric pulse, generated by photosensors and an amplifier as the rotor arm crossed the red probe light, was recorded (Figure S4). In total, 10 rotors (flies) for each sex were monitored simultaneously and tests for females and males were performed separately. Adults aged 15–20 days post-eclosion were used (Table S1). Prior to attachment to flight mill rotors, each fly was immobilized using carbon dioxide anesthesia [22]. Individuals were placed in 5-mL plastic centrifuge tubes, exposed to CO_2_ for approximately 5–10 s, and removed once immobile. Each fly was then affixed to the rotor via adhesive (G17, Konishi Co., Osaka, Japan; Bond, Konishi Co., Osaka, Japan) at the dorsal thorax. After recovery, flies that did not flap or exhibited abnormal movement were excluded from the experiment.

After mounting the rotor with an insect on the flight mill system, rotor revolutions were recorded for 2 h (see Table S3 for details). Periods with fewer than four revolutions per five seconds (equivalent to fewer than eight pulses) were considered non-flight, and total flight time was calculated by summing periods with eight or more pulses. This threshold was used because the rotors exhibited slight movement due to weak air currents within the incubating room, even when flies were not actively flying. The experiment was conducted at six temperature settings (9, 12, 15, 18, 21, and 24 °C), with 10 males and 10 females per temperature.

Temperature and relative humidity were recorded throughout the tests using thermohydrometers (TR72A2, T&D Co., Matsumoto, Japan).

### 2.4. Long-Duration Flight Test

A preliminary backward trajectory analysis indicated that adult *B. dorsalis* must sustain its flight for at least 24 h to migrate overseas from possible source areas of neighboring subtropical region in East Asia to the Kyushu District (Figure S5). To assess maximum flight duration, a 24-h flight test was conducted using the flight mill system.

Based on results from the takeoff observation (see below), two test groups were established: one starting at 10:00 and the other at 18:00 local time. Adults reared under 12L:12D and aged 8–17 days post-eclosion were used (Table S1). Room conditions were maintained at 21 °C with a 12L:12D photoperiod (light on from 6:00 to 18:00). To prevent dehydration during prolonged tethered flight, relative humidity was kept high using a humidifier (BE-J001, Zhongshan Zhuoweilai Electric Appliance Co., Ltd., Zhongshan, China).

Flies were mounted as described above, and rotor revolutions were recorded for 24 h. Flight time was calculated using the same criteria as described in the previous section. The 24-h flight test was conducted five times—three replicates starting at 10:00 and two at 18:00 local time—using 10 males and 10 females per replicate (totally 20 rotors were available at a single test). Thus, a total of 30 males and 30 females were tested for the 10:00 start, and 20 males and 20 females for the 18:00 start. The smaller sample size in the latter group was due to a limited supply of healthy individuals available at that time.

The fruit fly adults, females and males, used in this experiment were reared in large mixed-sex cages since post-eclosion. To assess the potential effect of mating status and ovarian maturity on female flight duration, the presence of sperm masses and mature eggs were recorded following the flight mill test. Ovaries and spermathecae were dissected from the female abdomen in water using tweezers under a stereo microscope (Leica Zoom TM2000, Leica Microsystems Inc., Wetzlar, Germany) at 4× to 40× magnification. Spermathecae were placed on a glass slide and gently crushed between the slide and a cover glass. Sperm masses within the spermathecae were then observed under a compound microscope (Olympus CH-2, Olympus Co., Tokyo, Japan) equipped with a phase contrast condenser unit at 400× magnification.

Additionally, in the 21 °C group described in Section 2.3, a preliminary test was conducted to measure male flight duration over a 14-h period. The experimental conditions were identical to those of the long-duration flight test, except for the photoperiod; specifically, the light remained on throughout the entire period.

Temperature and humidity were recorded during all the tests using the thermohydrometers (TR72A2, T&D Co., Matsumoto, Japan).

### 2.5. Statistical Analysis

All analyses were performed using R 4.4.3 (R Development Core Team, https://www.r-project.org/).

In Section 2.2, the effect of time on the number of individuals reaching the top of the cage was analyzed using Fisher’s exact test with multiple comparisons, implemented via the *fisher.multcomp* function in the RVAideMemoire package [23], with Holm correction.

To compare times taken to reach the top between local time groups (Section 2.2), and to compare total flight times between temperature groups (Section 2.3), we used the exact Wilcoxon-Mann-Whitney test. Holm’s correction was used to adjust for multiple comparisons. Given the small sample sizes and non-normal distributions, non-parametric methods were deemed appropriate.

In Section 2.3, nonlinear regression analysis [24] was performed using *nls* function to determine a low-temperature threshold at which *B. dorsalis* ceases flight activity. A sigmoid model of the form [25]

$$y=\frac{A}{1+e^{-k\left(x-x_{0}\right)}}$$

was fitted to the total flight time data, where *x* and *y* represent air temperature and total flight time, respectively. The constants *A*, *k*, *x*_0_ represent the asymptotic maximum flight time, the flight activity increasing rate, and the inflection point of air temperature, respectively. This model was selected because it effectively captures the relationship between air temperature and flight time. Initial parameter estimates were set as follows: *A* = maximum observed flight time, *k* = 1, *x*_0_ = median of air temperatures across test sections. The flight cessation temperature was estimated as the inflection point *x*_0_ of the fitted model.

The effect of sex on total flight times at various temperatures in Section 2.3 and Section 2.4 were tested with exact Wilcoxon-Mann-Whitney test. In Section 2.4, the effect of starting time on total flight times was tested using exact Wilcoxon-Mann-Whitney test, implemented via the coin package [26].

## 3. Results

### 3.1. Outdoor Takeoff Experiment

**Figure 1:**
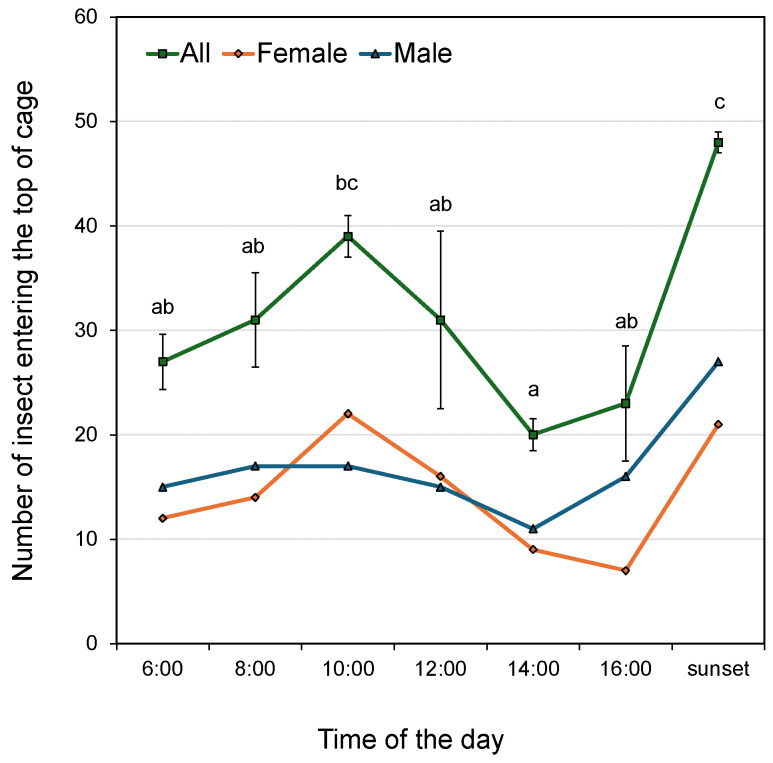
Average number of *Bactrocera dorsalis* individuals entering into the top section of the cage, observed at 2-h intervals. The green line represents the average number of individuals (both sexes), while the orange and blue lines represent females and males respectively. Error bars indicate standard deviations. Different letters above the data points indicate statistically significant differences (Fisher’s exact test, *p* < 0.05).

The number of *B. dorsalis* individuals that reached the top of the cage within 5 min after release exhibited two distinct peaks around 10:00 and sunset (Figure 1). In contrast, fewer individuals reached the top around 14:00 and 16:00. Among females, more than 20 individuals reached the top around 10:00 and sunset, whereas fewer than 10 did so around 14:00 and 16:00. Among males, the highest number (27) was recorded around sunset, while the lowest (11) occurred around 14:00.

A multiple comparison test of the total number of individuals (both sexes) reaching the top of the cage at each time point revealed statistically significant differences between the following pairs: 6:00, 8:00, 12:00, 14:00 or 16:00 vs. sunset, and 10:00 vs. 14:00 (Fisher’s exact test, *p* < 0.05).

**Figure 2:**
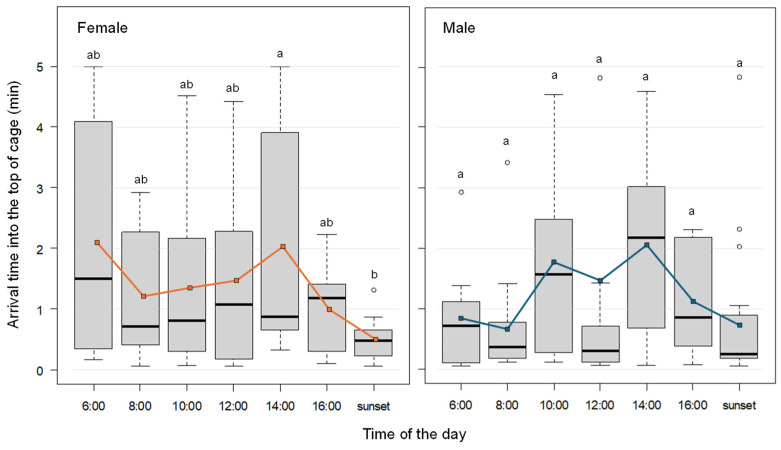
Arrival time for *Bactrocera dorsalis* individuals flying into the top section of cage at each takeoff time (average values shown as colored lines). The white circles in the boxplots indicate outliers. The left and right panels show data for females and males, respectively. Different letters above the box plots indicate statistically significant differences (exact Wilcoxon-Mann-Whitney test, *p* < 0.05).

The mean arrival time to the top of the cage was shortest around sunset for both sexes, when many fruit flies took off and ascended rapidly (Figure 2, Table S2). In contrast, fruit flies took longer to reach the top around 14:00. Among females, the shortest average arrival time (0.5 min) was observed around sunset, while longer times (exceeding 2 min) were observed around 6:00 and 14:00. For males, the shortest average times (0.5 min) were recorded around 6:00, 8:00, and sunset, with the longest time around 14:00 (exceeding 2 min).

A multiple comparison test indicated the medians of the arrival time between 14:00 and sunset for female were significantly different, while no significant difference was found between other time point pairs (exact Wilcoxon-Mann-Whitney test, *p* < 0.05). For males, no significant differences were found across time points (exact Wilcoxon-Mann-Whitney test, *p* > 0.05).

### 3.2. Low-Temperature Response Test

**Figure 3:**
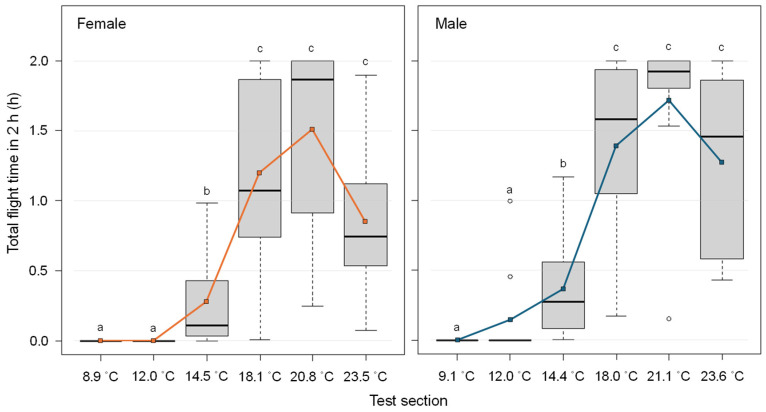
Total flight time of *Bactrocera dorsalis* individuals on 2-h flight mills under different temperature conditions. The horizontal axis indicates the average temperature (see Table S3). The white circles in the boxplots indicate outliers. The left and right panels show data for female and male, respectively. The orange and blue lines represent the mean total flight times for females and males, respectively. Different letters above the box plots indicate statistically significant differences (exact Wilcoxon-Mann-Whitney test, *p* <0.05).

Total flight times peaked at approximately 21 °C, with females averaging 1.5 h and males 1.7 h and decreased toward 9 °C, at which no individuals exhibited flight activity (Figure 3, Table S3). At around 24 °C, both the sexes showed shorter total flight times compared to those at 21 °C. The medians of total flight time were significantly different for both sexes between the following temperature pairs: 9 °C vs. 15–24 °C, 12 °C vs. 15–24 °C, and 15 °C vs. 18–24 °C (exact Wilcoxon-Mann-Whitney test, *p* < 0.05). No significant differences in the medians of total flight time between sexes were observed at any temperature setting (exact Wilcoxon-Mann-Whitney test, *p* > 0.05).

The fitted sigmoid models of the total flight time for both sexes were presented in Table 1 and Figure S6. Flight cessation temperatures were estimated as the inflection points of the respective models: 16.5 °C for females and 16.2 °C for males.

### 3.3. Long-Duration Flight Test in a 24-h Period

**Figure 4:**
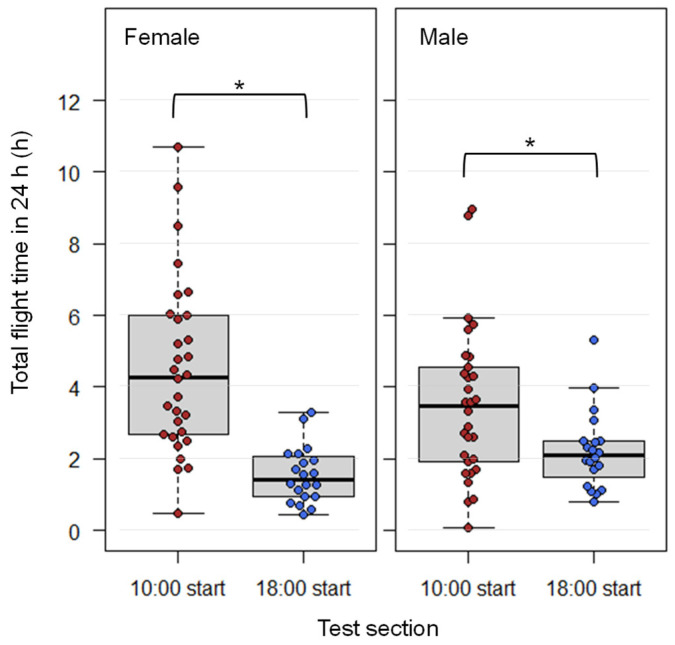
Total flight time of *Bactrocera dorsalis* individuals in the 24-h flight mill tests starting at 10:00 and 18:00. The left panel shows data for females, and the right panel for males. Colored dots represent the total flight time of each fruit fly. Asterisks indicate statistically significant differences (exact Wilcoxon-Mann-Whitney test, *p* < 0.05).

Some individuals (13.3% of the females, 6.6% of the males) exhibited their total flight times exceeding 7 h (Figure 4, Table S4). The longest individual flight durations were 10.7 h for females and 9.0 h for males. Additionally, a preliminary flight mill test conducted with males over a 14-h period revealed that one male reached a total flight time of 12.5 h (Figure S7).

The medians of the flight durations between the 10:00 and 18:00 start groups were significantly different for both sexes (exact Wilcoxon-Mann-Whitney test, *p* < 0.05). A significant difference in median flight duration between sexes was observed in the 10:00 group, but not in the 18:00 group.

Post-experiment examination of female ovaries revealed that all females tested lacked mature ovarian eggs, indicating reproductive immaturity. Furthermore, no sperm masses were observed in the spermathecae, confirming that none of the females had mated.

## 4. Discussion

### 4.1. Outdoor Takeoff Experiment

A bimodal peak was observed in the number of *B. dorsalis* individuals that spontaneously took off and ascended to the top of the cage, with the highest activity occurring around 10:00 and sunset. The former time could alter a little depending on the starting time and intervals of the experiment. A previous field trap survey conducted in late June in Yunnan Province, China, reported increased male captures between 8:00–9:00 and 18:00–20:00 local time [11]. Another survey in the same province indicated two peaks in male captures from early June to early July, occurring at 10:00 and 16:00 local time [13]. Similarly, in early August, in Taipei City, Taiwan, peak captures were observed between 6:00–10:00 and 16:00–18:00 local time [12]. The timing of peak activity observed in the present study aligns closely with these previous reports. Although concerns have been raised that trap captures may reflect rhythmic responsiveness to methyl eugenol rather than actual flight activity, the present results suggest that these patterns indeed represent diurnal changes in flight behavior. Furthermore, because females are not attracted to methyl eugenol, previous studies have not reported their diurnal flight activity. This study demonstrates that females also exhibit a bimodal flight activity pattern like that of males.

The time required to reach the top of the cage was shortest around sunset and longest at 14:00 for both sexes. However, shorter ascent times did not always coincide with periods of high activity; for example, at 10:00, males exhibited longer average ascent times despite elevated flight activity. The factors that stimulate flight behavior in the morning and around sunset remain unclear. However, observations indicate that as a diurnal species, this insect exhibits increased activity during the morning, as reflected in high trap capture rates. Furthermore, they are known to mate actively at dusk. These characteristics may relate to flight behavior.

On a calm, sunny day, the sun warms the Earth’s surface at sunrise, and the sensible heat flux from the ground gradually breaks down the surface inversion layer formed during the night. As the surface temperature rises, pockets of air warmer than their surroundings—known as thermals—gain buoyancy and begin to ascend. As the solar altitude increases and surface heating approaches its peak, convection becomes more active. Around 10:00, this vigorous convection causes updrafts to extend forcefully upward, thickening the “mixed layer” where upper and lower air are intensely stirred. Therefore, if fruit flies were to take flight at this time, they could potentially gain altitude by utilizing such updrafts.

However, under meteorological conditions where strong southwesterly monsoons or low-level jets drive long-distance insect migration to Japan, winds with strong horizontal components likely prevail even in the lower atmosphere of the source regions. In such cases, the flies may initiate their migration by riding these prevailing winds during their peak takeoff times.

Given that the present experiment was conducted from late October to early November, seasonal shifts in sunrise and sunset times should be considered when modeling peak flight activity for early summer of the main migration season. Regarding the outdoor experiments conducted from October to November, the photoperiod in the study site (Taichung city) between October 23 and November 4 ranged from 11 h 10 min to 11 h 30 min. In contrast, the photoperiod during the migratory season (late May) is 13 h 20 min, which is approximately 2 h longer. Additionally, sunrise occurs about 1 h earlier, and sunset occurs later during the migratory season compared to the experimental period.

### 4.2. Low-Temperature Response Test

Both sexes exhibited the longest flight durations at 21 °C. This finding is consistent with Makumbe et al. [15], who reported that laboratory-reared flies from South Africa (18 generations) flew the longest distances at 20 °C. Similarly, laboratory-reared flies from Taiwan in the 1930s did not initiate flight below 20 °C and remained inactive at 13–14 °C [14]. In the present study, only two males flew at 12 °C, while all other individuals ceased flying, supporting previous observations.

Based on sigmoid model fitting, flight cessation temperatures were estimated as 16.5 °C for females and 16.2 °C for males. This difference may reflect the tendency of males to fly slightly longer than females across all tested temperatures, with several males still flying at 12 °C. For trajectory modeling, a flight cessation temperature of 16.2 °C is recommended for *B. dorsalis*, as males are capable of sustained flight at slightly lower air temperatures.

At 24 °C, both sexes exhibited shorter average flight durations compared to those at 21 °C. A similar pattern was also reported [15]. Although the sample size per temperature group in our experiment was limited to 10 individuals, the results reliably capture a key aspect of the species’ flight behavior under low-temperature conditions.

### 4.3. Long-Duration Flight Test

Fruit flies in the 10:00 start group exhibited longer flight durations than those in the 18:00 start group. This difference is attributed to reduced flight activity during the dark period (18:00–6:00) compared to the light period (6:00–18:00) (Figure 4 and Figure S8). These findings suggest that darkness may suppress flight activity, and individuals initiating flight in the morning may be capable of long-distance movement during the daylight hours. Nevertheless, many individuals continued intermittent flight during the dark period (Figure S8), suggesting that this species potentially possesses the capacity for sustained flight during the night under certain conditions—such as overseas migration—where landing is not an option and nocturnal flight should be considered in models of long-distance migration across oceanic barriers.

In this study, six individuals flew for more than seven hours, one exceeded 10 h, suggesting that a subset of *B. dorsalis* individuals possess the capacity for long-distance flight. Note that we do not claim that the species generally possesses a “capacity for long-distance flight”. Instead, we state that our long-duration flight tests indicate that certain individuals within the population exhibit long-duration flight capabilities. This assertion is supported by our experimental results, as well as the fact that such individuals have immigrated to and been recently trapped in western Japan. Although no individuals reached the 24-h threshold estimated by backward trajectory analysis as necessary for overseas migration, the observed flight durations exceed those reported in previous flight mill studies, which primarily used laboratory-reared flies with potentially reduced flight capacity. The use of wild-origin individuals in this study provided more accurate data to reveal the flight characteristics.

Regarding the flight speed, we calculated the speeds from the rotation counts of the flight mills. The results showed average speeds of 1.4 m/s for females and 1.5 m/s for males at both 21 °C and 24 °C, with the fastest individuals of both sexes reaching 2.0 m/s. However, the previous study [21] reported that the free-flight speed of this species ranges from 3.0 to 4.0 m/s. Our experimental results were approximately half of those reported in the literature. We attribute this discrepancy to the mechanical load (weight) of the flight mill rotors, which likely reduced the insects’ speed, suggesting that the flight mill may not provide an accurate measure of maximum flight speed. Consequently, we believe it is more appropriate to refer to the direct measurement study when selecting parameters for migration estimation or flight simulations.

### 4.4. Sex-Based Differences in Flight Time

Regarding sex-based differences in flight duration, it is reported that mated females of Vietnamese origin (4–11 laboratory generations) flew significantly longer than males [27]. Similar trends have been observed in related species, including *Z. cucurbitae* [16], *B. latifrons* [17], and the guava fruit fly *Bactrocera correcta* (Bezzi) [28]. Conversely, no significant sex-based differences were found in Chinese-origin oriental fruit flies (approximately 20 laboratory generations) [29] or in Hawaiian populations [30].

In the long-duration flight test conducted in this study, females appeared to fly longer than males in the 10:00 start group, although the difference was not statistically significant. In contrast, males flew significantly longer than females in the 18:00 start group. In the 2-h flight test, males tended to fly longer than females across temperature groups, but no significant differences were detected.

Given that wild-origin individuals were used in this study, direct comparisons with previous studies are limited. However, the results suggest that sex-based differences in flight performance may be minimal overall and that environmental conditions could play a more influential role. Importantly, because females are of greater concern in quarantine contexts, their ability to fly as long as or longer than males warrants attention.

### 4.5. Effect of Mating Status on Female Flight Ability

This study did not directly assess the effect of mating status on female flight ability, as all females used were unmated and reproductively immature. It is reported no significant difference in flight duration between mated and unmated females in Vietnamese-origin oriental fruit flies (4–11 laboratory generations) [27]. In contrast, it was found that 30-day-old unmated females of *Z. cucurbitae* flew longer than mated females of the same age [31]. Further research is needed to determine whether mating status affects flight performance of *B. dorsalis* under varying conditions.

## 5. Conclusions

Flight experiments using wild first-generation *B. dorsalis* from Taiwan revealed that peak flight activity occurs at 10:00 and sunset, and that the longest flight durations were observed at 21 °C. Flight cessation temperatures were estimated to be between 16.2 °C and 16.5 °C. Additionally, some individuals flew for more than 7 h, with one individual exceeding 10 h, indicating the species’ potential capacity for long-distance flight.

These findings provide fundamental insights into the flight characteristics of *B. dorsalis* associated with long-distance migration and offer essential parameters for flight simulation models. This contributes to improving the accuracy of migration predictions and enhancing pest management strategies.

## Figures and Tables

**Table 1:** Estimated parameters of sigmoid models fitted to the total flight time data (Figure S5) using nonlinear least square (*nls*() in R).

Parameter †	Female				Male			
	Estimate	Std. Error	*t* Value	*p* Value	Estimate	Std. Error	*t* Value	*p* Value
*A*	1.56	0.20	7.781	<0.001	1.78	0.19	9.277	<0.001
*k*	0.78	0.27	2.877	0.006	0.69	0.20	3.381	0.001
*x*_0_	16.53	0.76	21.815	<0.001	16.19	0.68	23.68	<0.001

† *A*, the asymptotic maximum flight time; *k*, the flight activity increasing rate; *x*_0,_ the inflection point of air temperature.

## Data Availability

Data supporting this study are available at http://datadryad.org/share/LINK_NOT_FOR_PUBLICATION/FZH4L3UodQAIFgIWOuOgeQX79MQWjkcdbOthJ3DnFlQ.

## Supplementary Materials

The following supporting information can be downloaded at: https://www.mdpi.com/article/10.3390/insects17020203/s1, Figure S1: Location map; Figure S2: Cage and release box used for the outdoor takeoff test; Figure S3: Flight mill system; Figure S4: Electronic circuit diagram of the flight mill system; Figure S5: Example of backward trajectory analysis for a trap catch in Kumamoto Prefecture in the Kyushu District on 26 May 2021; Figure S6: Sigmoid curves fitted to the total flight time of *Bactrocera dorsalis* females and males at different temperatures; Figure S7: Total flight time of *Bactrocera dorsalis* male in the 14-h flight experiment; Figure S8: Raw rotation data in the long-duration flight test; Table S1: Sample information; Table S2: Conditions of the outdoor takeoff test; Table S3: Conditions of the low-temperature response test; Table S4: Conditions of the long-duration flight test.
