# Supplementary material for: Flight Characteristics of Bactrocera dorsalis Associated with Long-Distance Migration
Source: Insects. 2026 Feb 14;17(2):203. doi: 10.3390/insects17020203 (PMC12940858; doi:10.3390/insects17020203)
Supplement: Supplementary file 1 [file insects-17-00203-s001.zip › insects-4035873-supplementary.pdf]

Supplementary materials for "Flight characteristic of *Bactrocera dorsalis* associated with long-distance migration"

Naoya Hidaka, Kohei Nishiya, Yudai Masuoka, Akiya Jouraku, Yong-Jun Yang, Chia-Jung Ho, Yu-Bin Huang, Akira Otuka

The supplementary materials present a location map, schematics of takeoff cage, and flight mill system, detailed data analyses and supplementary tables.

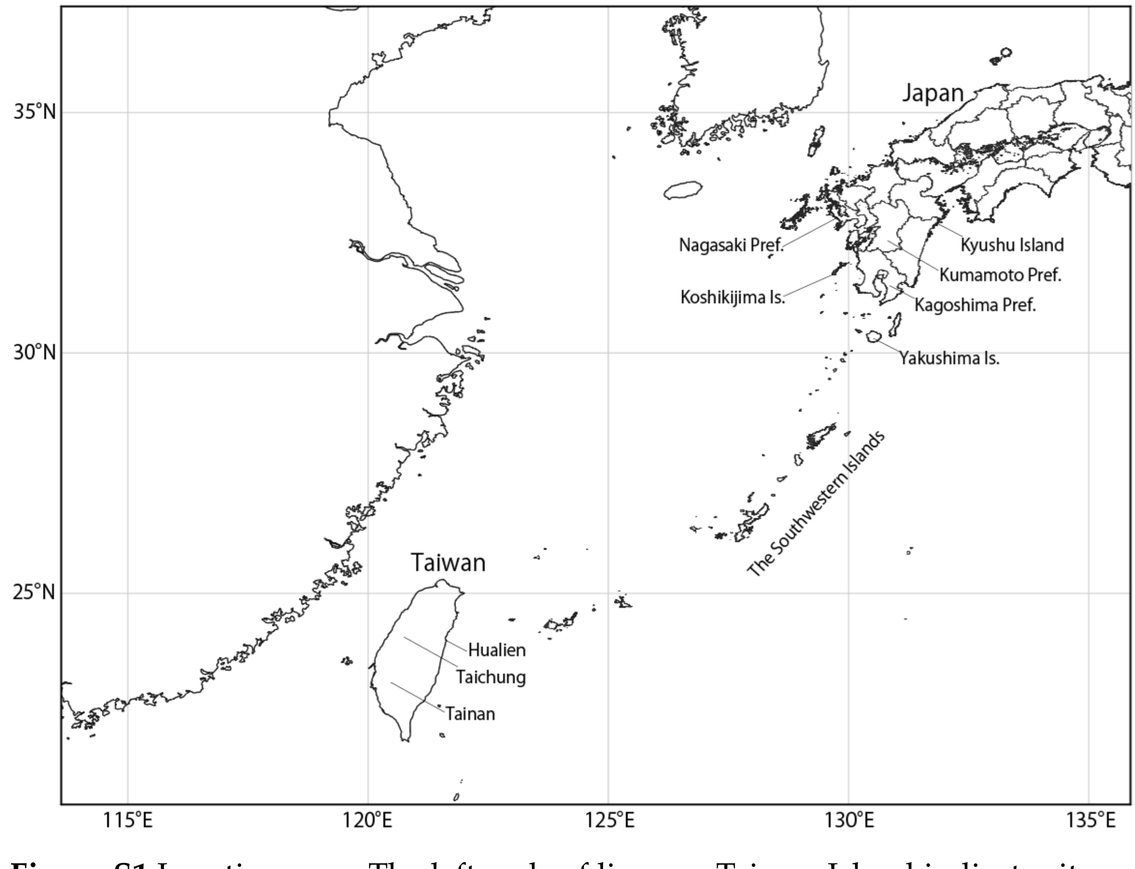

Figure S1 Location map. The left ends of lines on Taiwan Island indicate sites of insect collection in three cities.

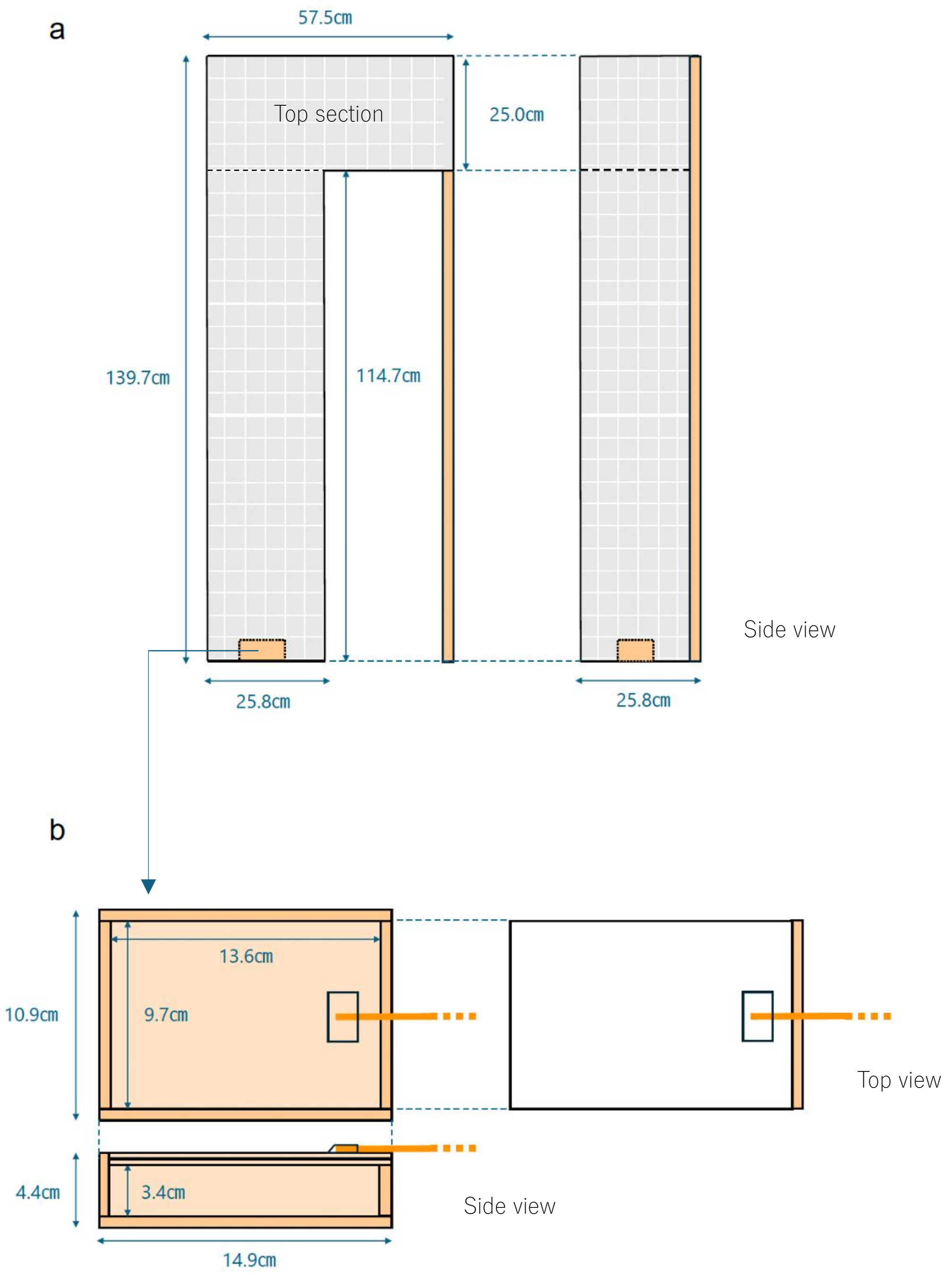

Figure S2 Cage and release box used for the outdoor takeoff test. **(a)** A vertically long cage **(b)** A release box placed on the bottom of the cage, from which 10 *Bactrocera dorsalis* males or females take off. To release the flies from the box, a transparent acrylic sliding top cover was quietly pulled by a string attached to the cover.

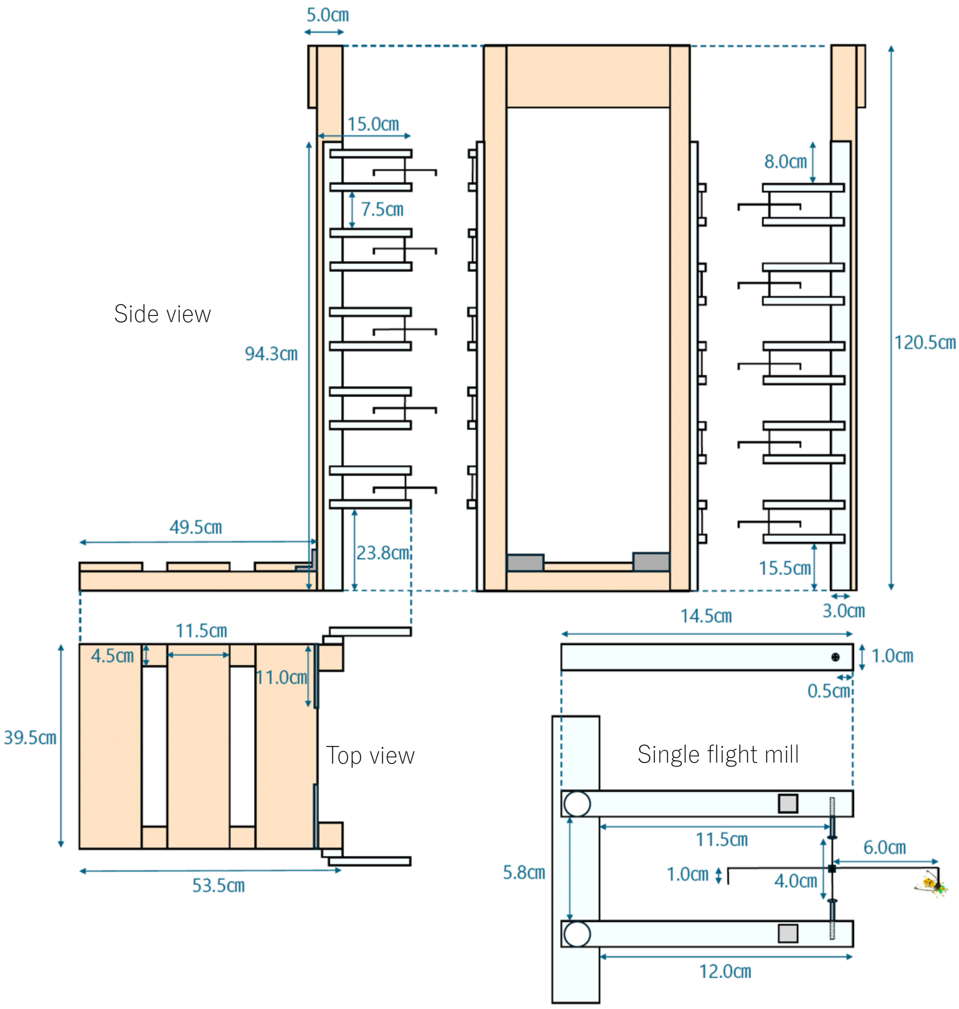

Figure S3 Flight mill system. The arm of a rotor (rotation radius: 60 mm) was made of a thin stainless-steel wire of 0.5 mm dia. with total length of 140 mm, whose both ends were bent downwards by 10 mm. The vertical axis is a 40-mm stainless-steel insect pin (Insect pins without heads No.00, Shiga Konchu Fukyu Co., Tokyo, Japan). The arm and the axis were vertically connected using a cubic piece of sponge rubber (approximately 5 mm on each side). The average weight of the rotor assembly was 93.7 ± 0.6 mg (n = 10). Light blue rods and light brown rods and plates were acrylic and wooden, respectively.

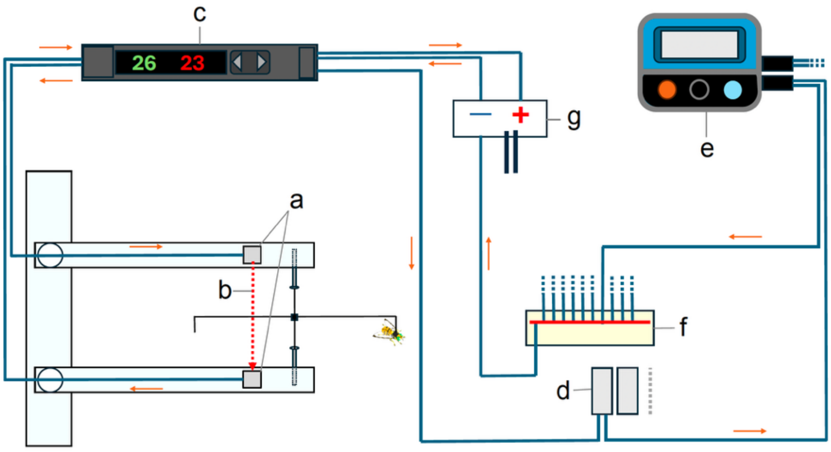

Figure S4 Electronic circuit diagram of the flight mill system. Orange arrows indicate the signal flow. **a:** Optical sensor. Each unit consists of a pair of LED emitter and receiver components. A signal is counted when the rotor interrupts the red LED beam; **b:** LED beam; **c:** Amplifier-electric pulse generator; **d:** Crimp terminal made of aluminum tube; **e:** Pulse logger; **f:** Breadboard used to unify the negative voltage lines; **g:** DC power supply. Rotor revolutions were counted every 5 sec using the optical sensor (FU-51TZ, Keyence, Osaka, Japan), amplified by the amplifier (FS-N11N and FS-N12N, Keyence, Osaka, Japan), and recorded by the pulse logger (LR8512, HIOKI Co., Ueda, Japan).

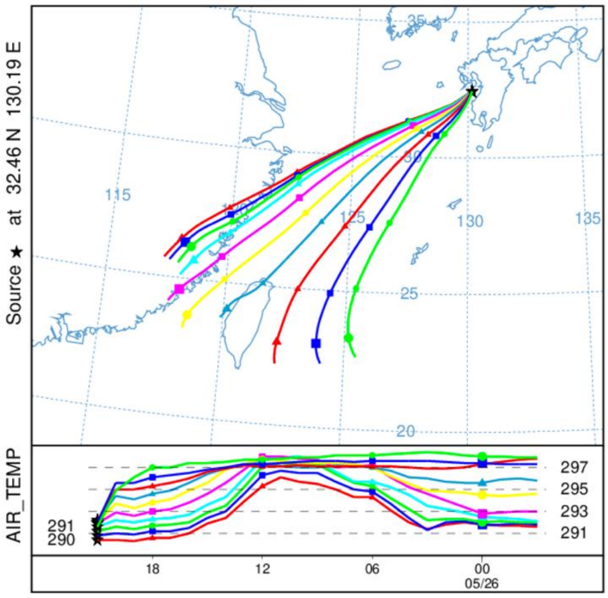

Figure S5 Example of backward trajectory analysis for a trap catch in Kumamoto Prefecture in the Kyushu District on May 26, 2021. The trajectories were

calculated using the HYSPLIT (Hybrid Single-Particle Lagrangian Integrated Trajectory) model developed by the National Oceanic and Atmospheric Administration (NOAA) (Draxler & Hess, 1997). The calculation conditions were as follows: flight initiation time: 21:00 UTC (6:00 JST); flight speed: 3.0 m/s (Hirabayashi et al., 2012); arrival flight altitude: from 100 to 1000 m at 100-m intervals shown in different colors; low temperature threshold: 16.2 °C. Air temperatures in K along the trajectories were plotted in the lower panel.

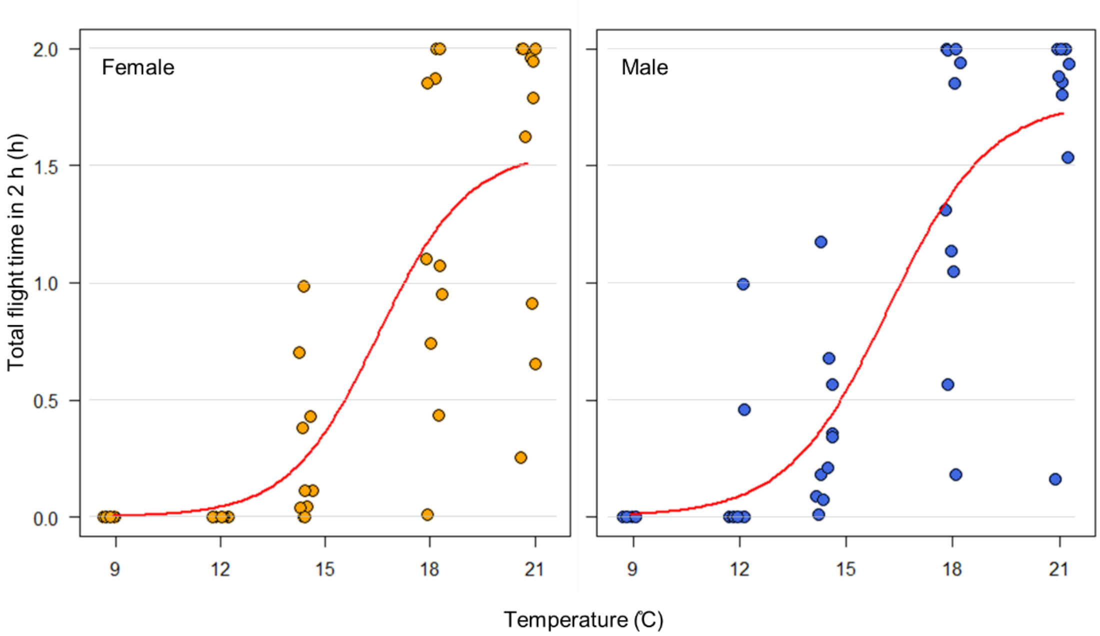

Figure S6 Sigmoid curves (red lines) fitted to the total flight time of *Bactrocera dorsalis* females (left) and males (right) at different temperatures below 21 °C in the 2-h flight mill test, using nonlinear least squares (*nls* function in R). Colored dot indicates the total flight time per individual.

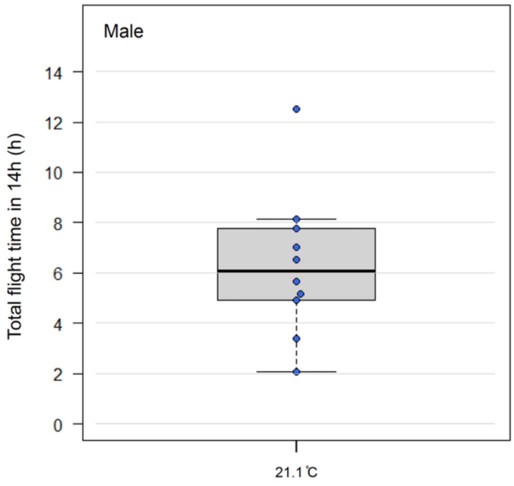

Figure S7 Total flight time of *Bactrocera dorsalis* male in the 14-h flight experiment. Dot indicates the flight time per individual. This experiment was preliminarily conducted before the long-duration flight test (24 h). The longest total flight time > 12 h was recorded by a male.

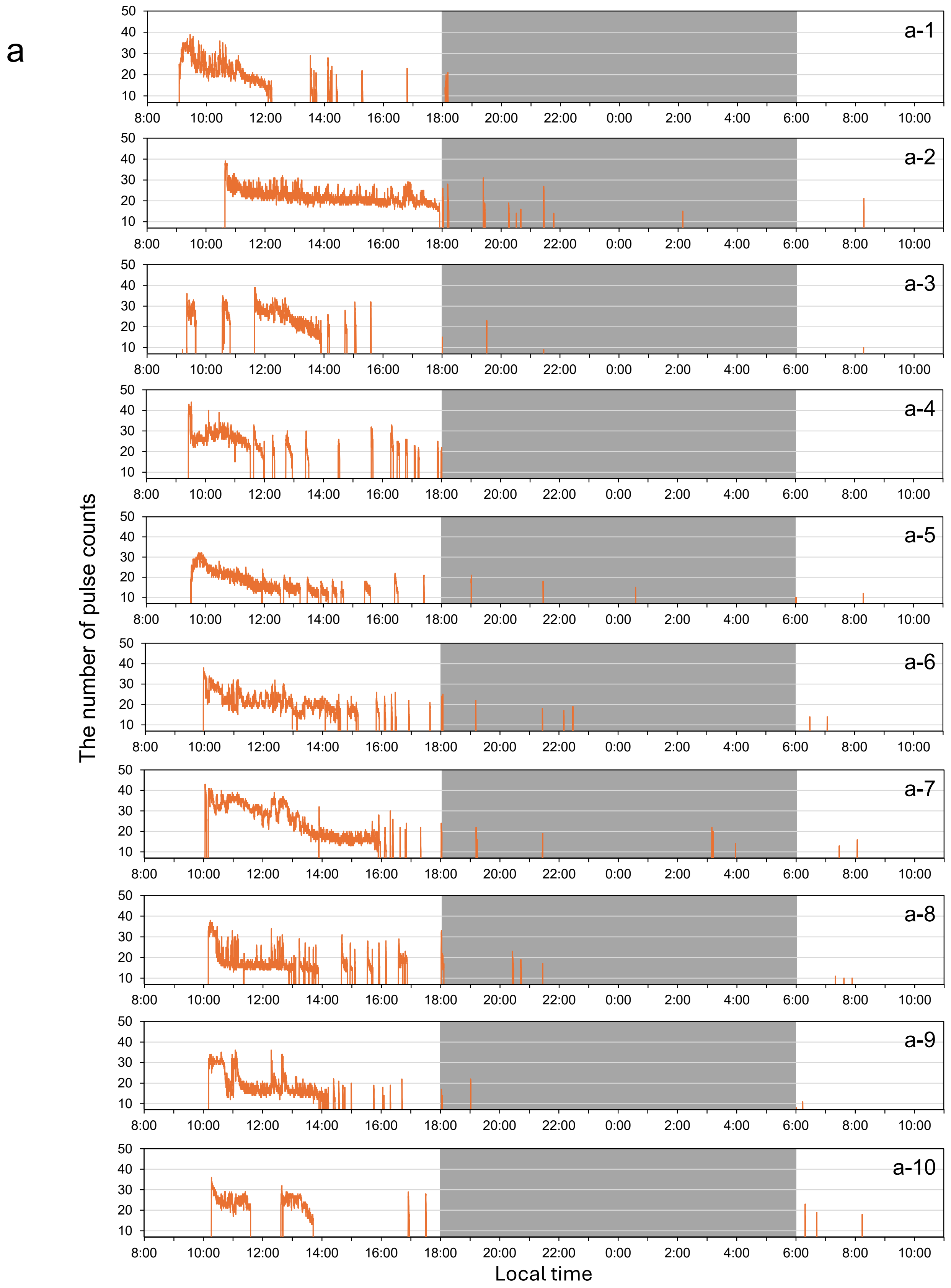

Figure S8 Raw rotation data in the long-duration flight test. The vertical and horizontal axes represent pulse counts per 5-second interval and local time, respectively. One rotation generated two pulse counts. Data segments exceeding eight pulse counts per 5-second were defined as flight zones. Panels **a** - **c** show data for females in the 10:00 start group; panels **d** - **f** for males in the 10:00 start group; panels **g** and **h** for females in the 18:00 start group; and panels **i** and **j** for males in the 18:00 start group. The gray-shaded area indicates the dark period.

Figure S8 (continued)

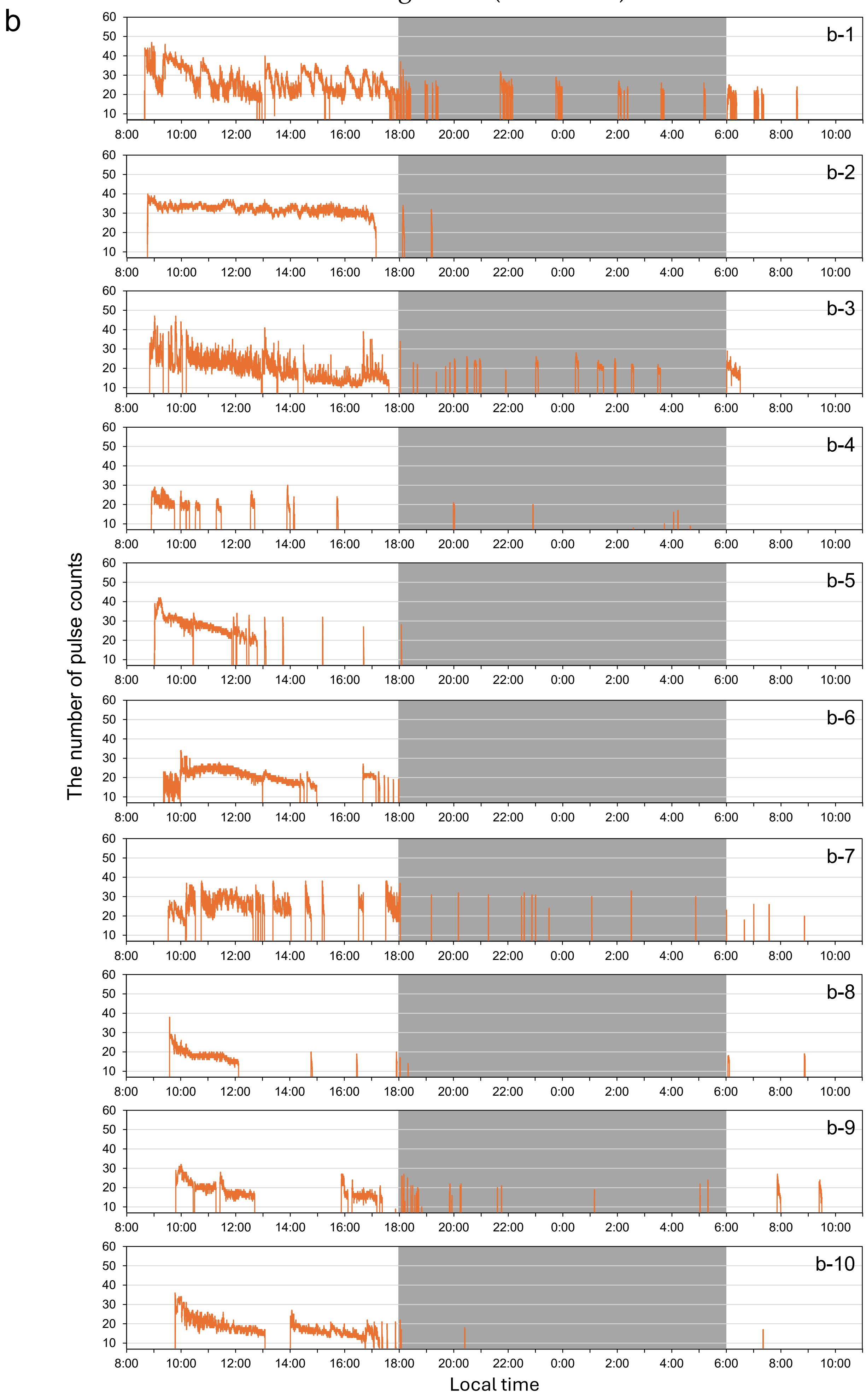

Figure S8 (continued)

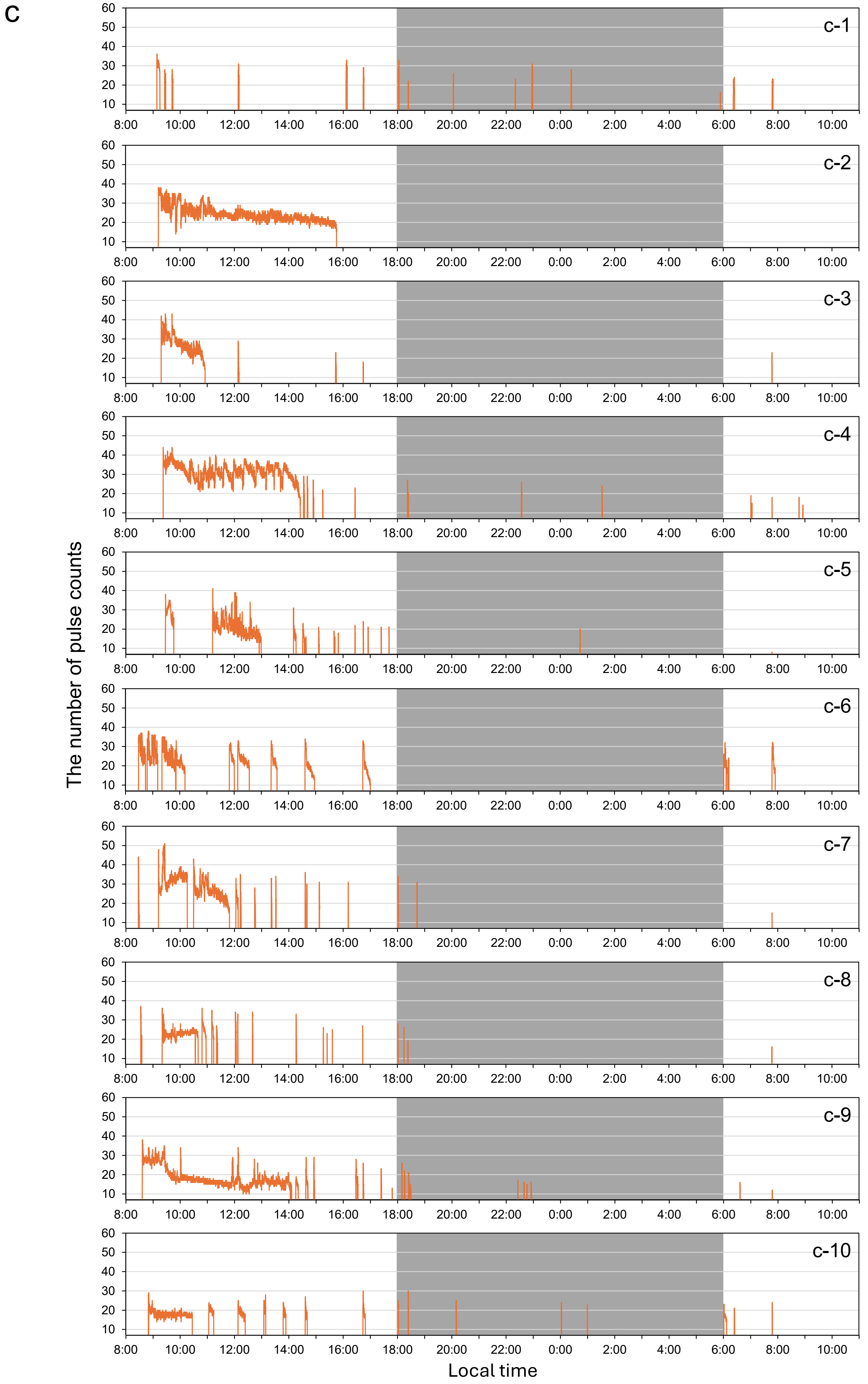

Figure S8 (continued)

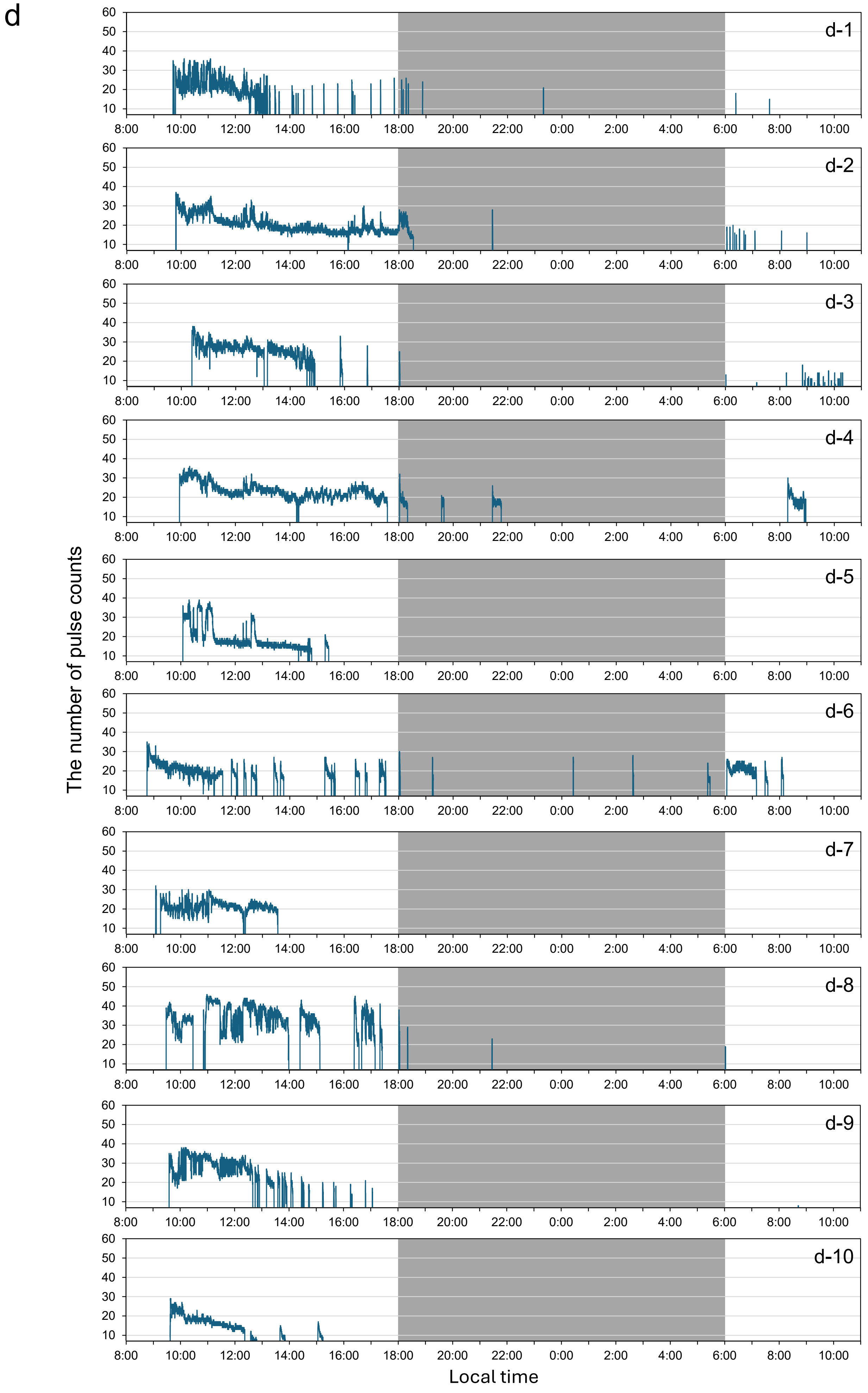

Figure S8 (continued)

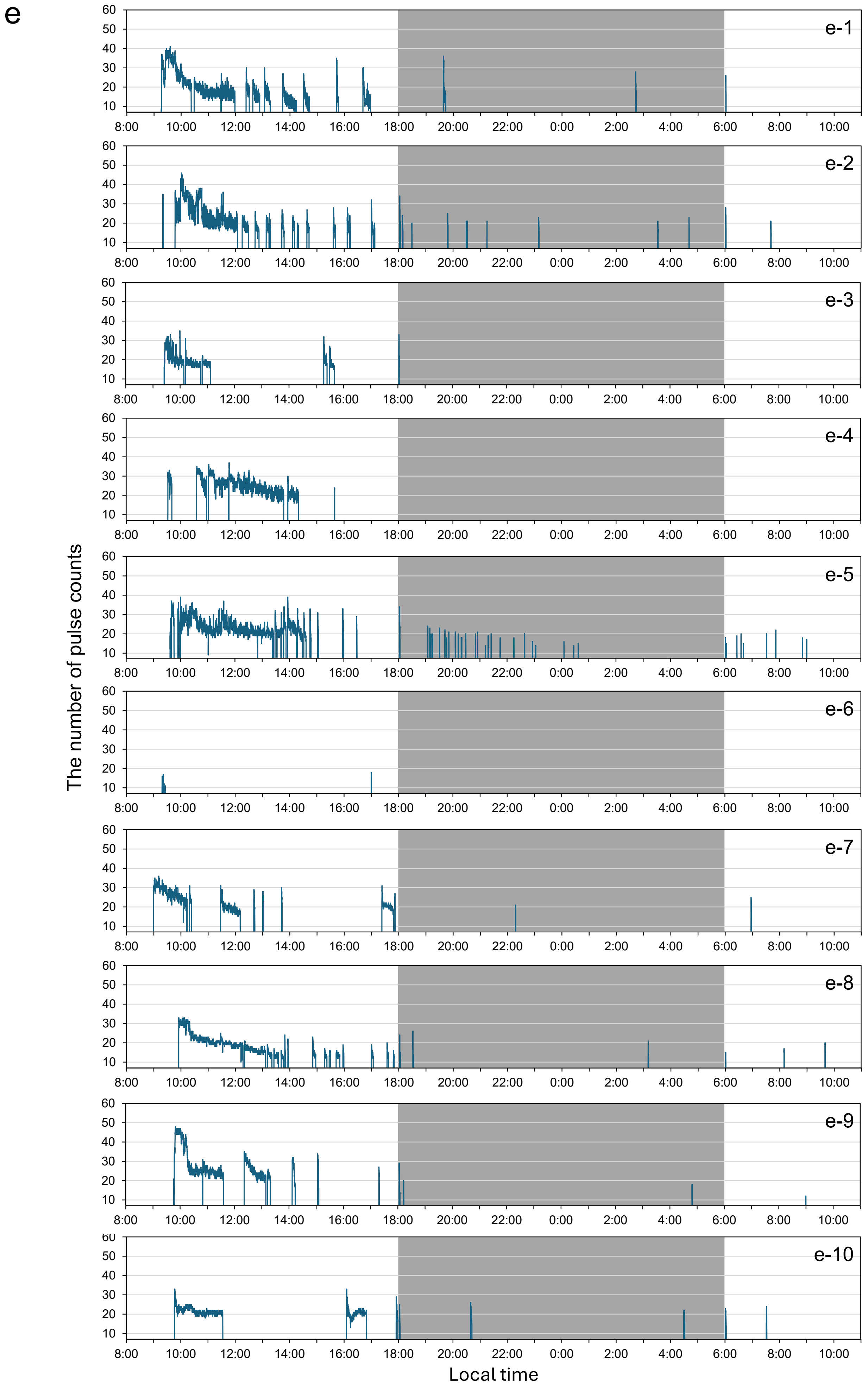

Figure S8 (continued)

f

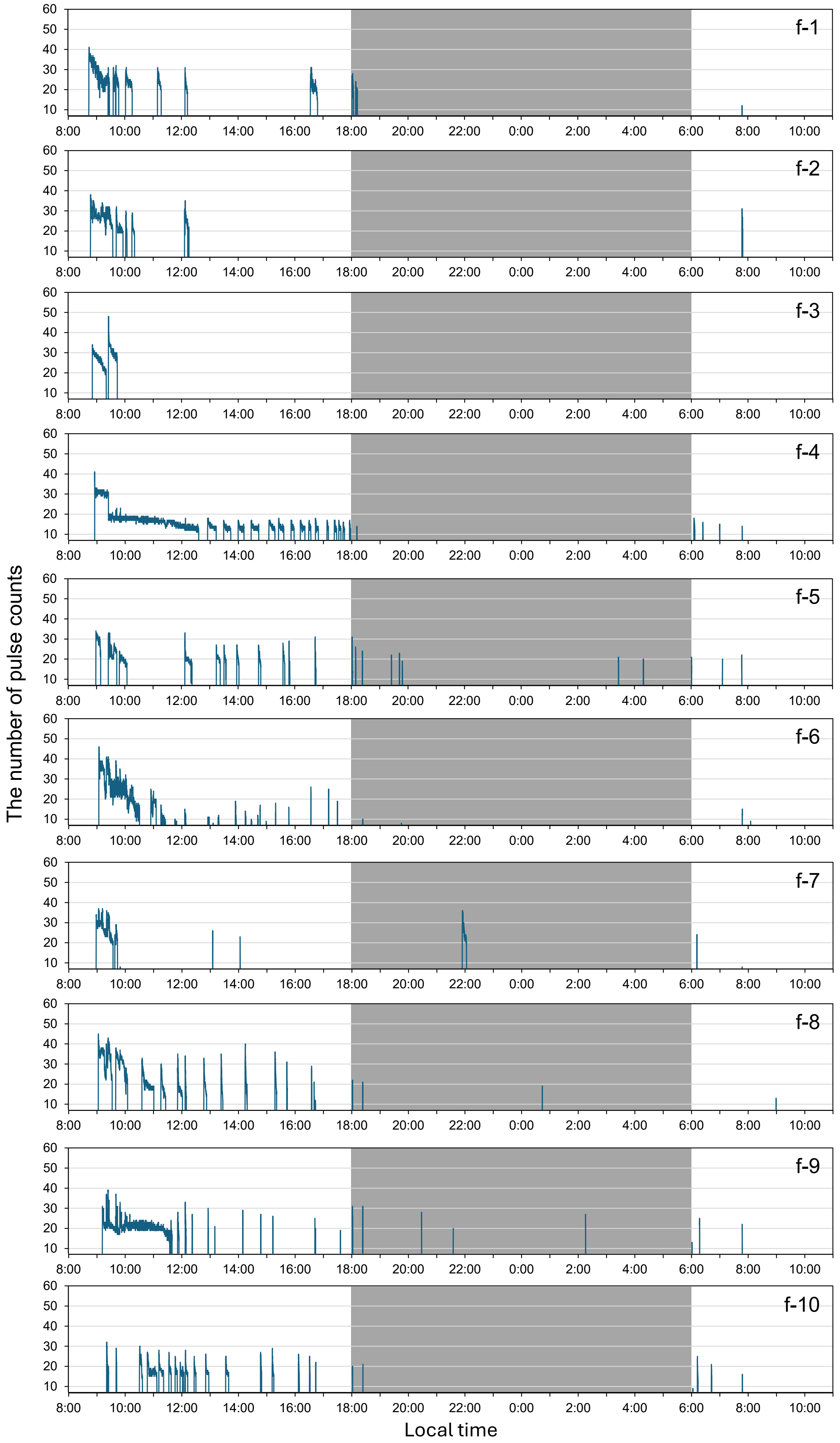

Figure S8 (continued)

g

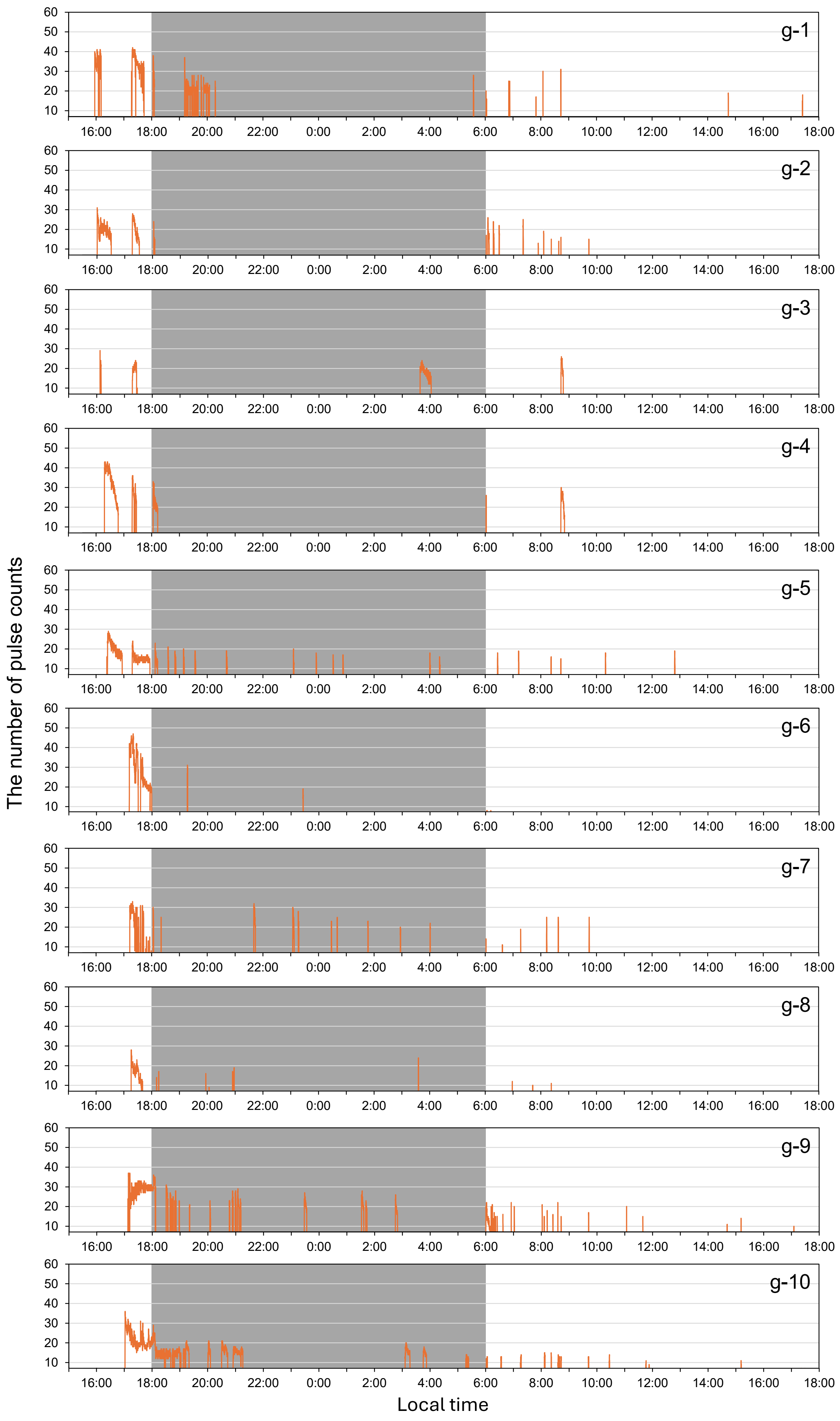

Figure S8 (continued)

h

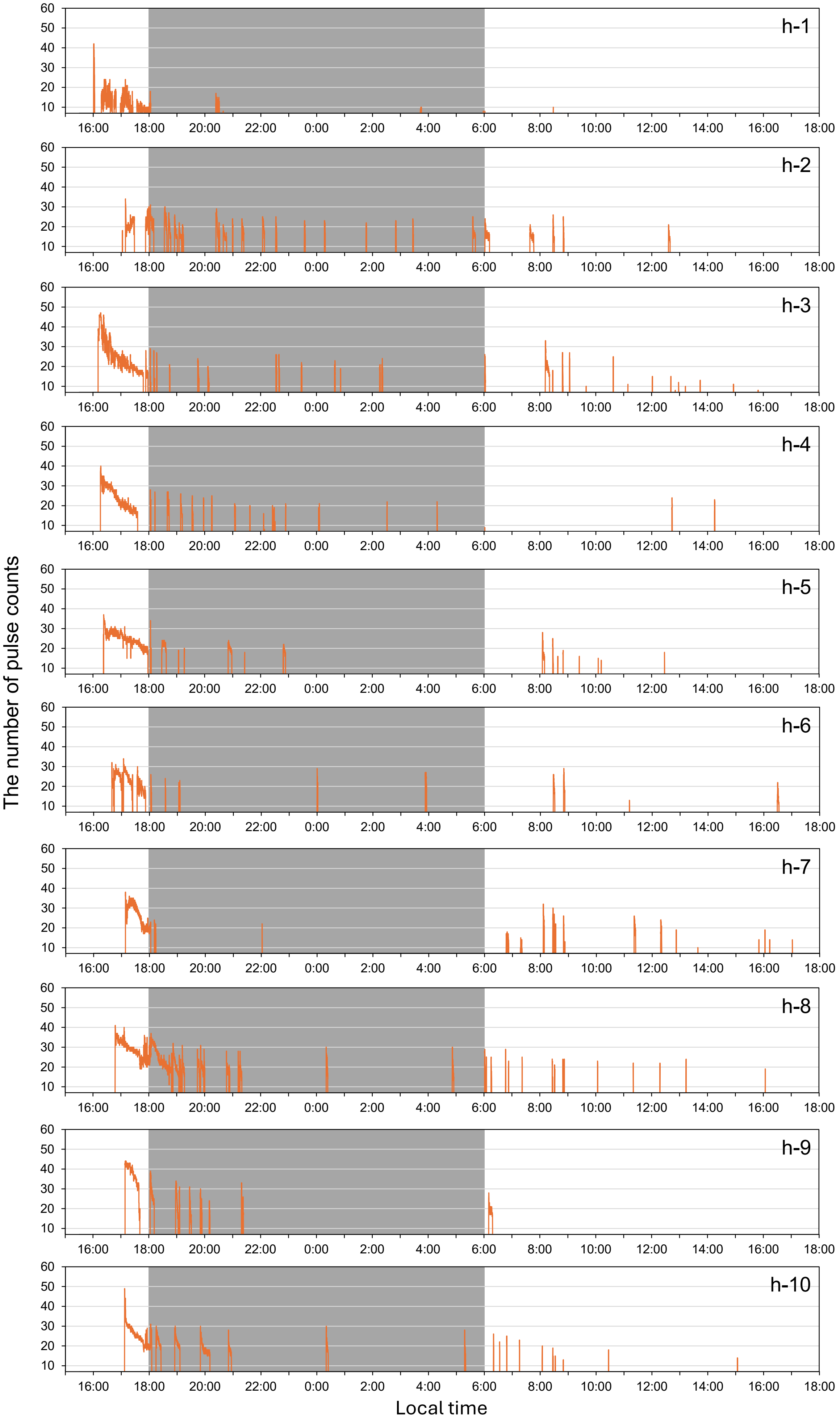

Figure S8 (continued)

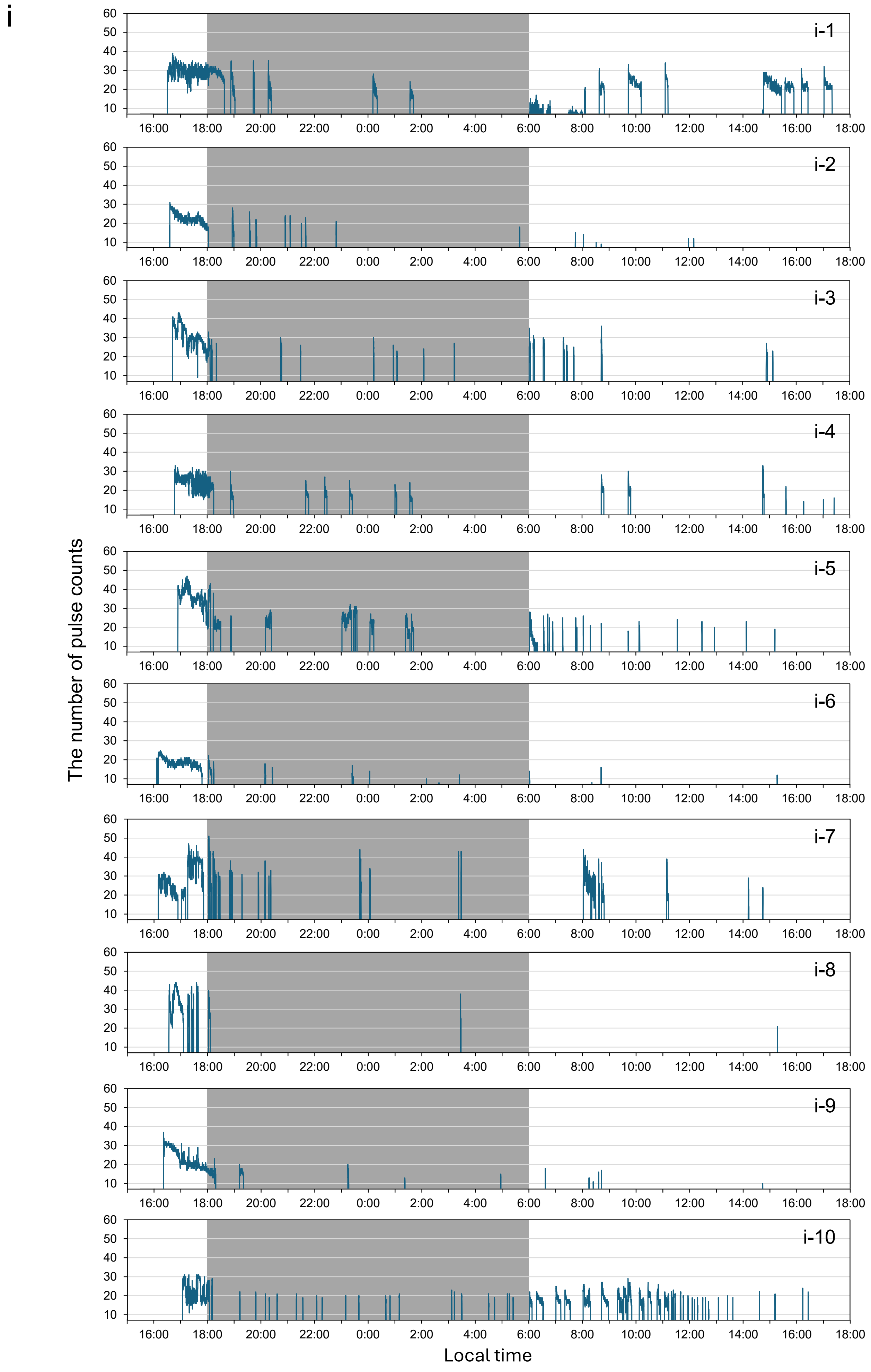

Figure S8 (continued)

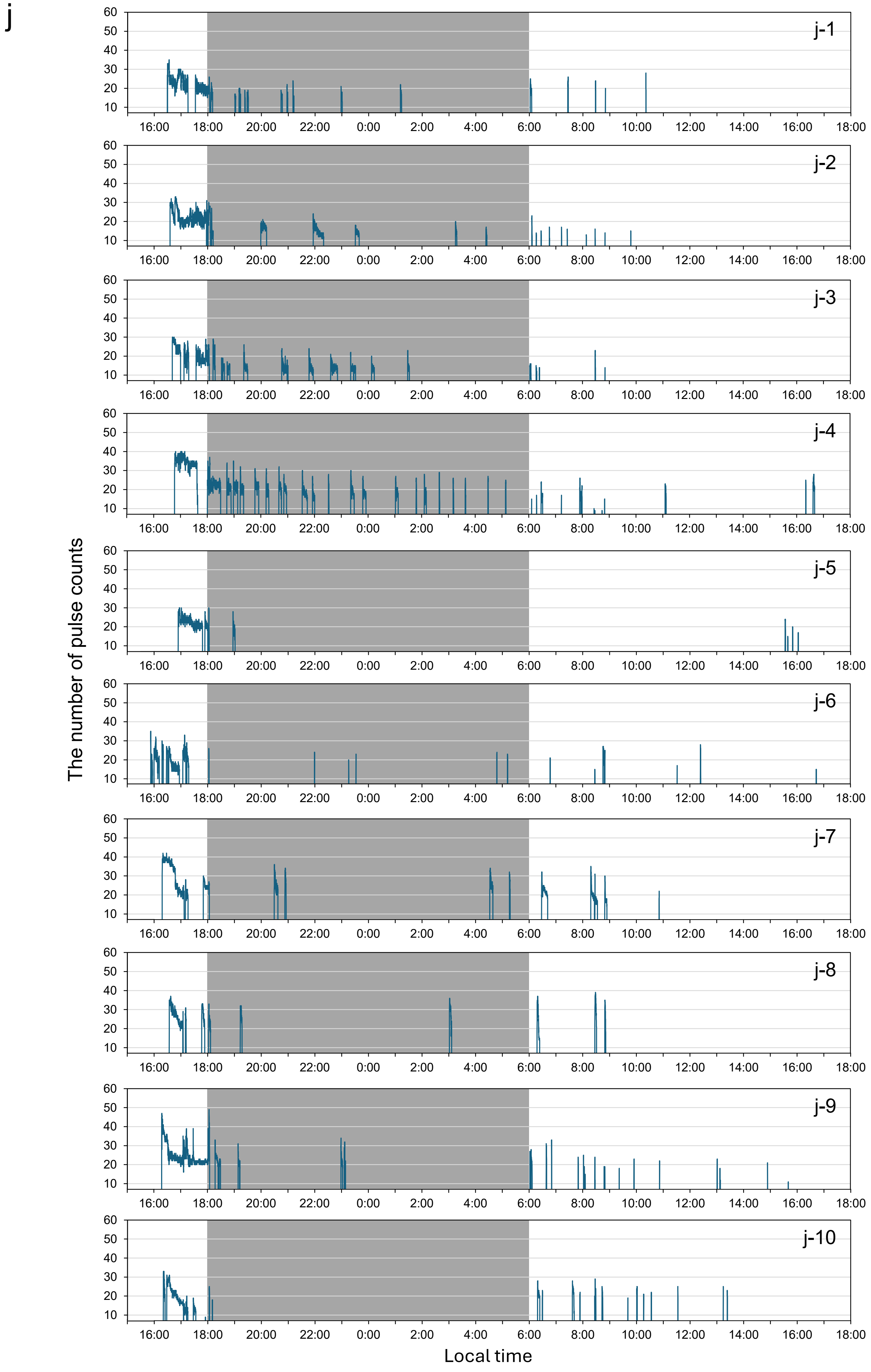

Table S1. Sample information.

Experimental type	Experimental date	Test section	Location of collecting infected fruits	Latitude, Longitude	Date of collecting infected fruits	Species of infected fruits	Date of hatching of adult insects	Age of adult insects
Outdoor takeoff experiment	23-Oct-24	6:00	Taichung City, Taiwan	24.036 ˚N,120.691 ˚E	25-Sep-24	Guava	8-Oct-24	15
		8:00						15
		10:00						15
		12:00						15
		14:00						15
		16:00						15
	24-Oct-24	6:00	Taichung City, Taiwan	24.036 ˚N,120.691 ˚E	25-Sep-24	Guava	9-Oct-24	16
		8:00						16
		10:00						16
		12:00						16
		14:00	Hualien City, Taiwan	24.041 ˚N, 121.604 ˚E	20-Sep-24	Pomelo	11-Oct-24	13
		16:00						13
		sunset						13
	25-Oct-24	6:00	Hualien City, Taiwan	24.045 ˚N, 121.602 ˚E	20-Sep-24	Pomelo	11-Oct-24	14
		8:00						14
		10:00						14
		12:00						14
		14:00	Taichung City, Taiwan	24.036 ˚N,120.691 ˚E	25-Sep-24	Guava	13-Oct-24	12
		16:00						12
		sunset						12
	4-Nov-24	sunset	Tainan City, Taiwan	23.287 ˚N,120.509 ˚E	9-Oct-24	*Citrus* sp.	27-Oct-24	8
	1-Nov-24	9 ˚C female	Taichung City, Taiwan	24.036 ˚N,120.691 ˚E	25-Sep-24	Guava	13-Oct-24	19
	2-Nov-24	9 ˚C male						20

Low-temperature response test	30-Oct-24	12 ˚C						17
	30-Oct-24	12 ˚C male						17
	28-Oct-24	15 ˚C						15
	28-Oct-24	15 ˚C male						15
	31-Oct-24	18 ˚C						18
	1-Nov-24	18 ˚C male						19
	29-Oct-24	21 ˚C						16
	29-Oct-24	21 ˚C male						16
	28-Oct-24	24 ˚C						15
	28-Oct-24	24 ˚C male						15
Long-duration flight test	27-May-25	10:00 start	Taichung City, Taiwan	24.032 ˚N, 120.694 ˚E	5-May-25	Peach	19-May-25	8
	30-May-25							11
	5-Jun-25							17
	28-May-25	18:00 start						9
	3-Jun-25							15

Table S2. Conditions of the outdoor takeoff experiment.

Date	Start time	End time	Air temperature (˚C)	Humidity (%)	Wind speed (m/s)	Weather
23-Oct-24	6:12	6:17	25.7	70.1	0.1	Sunny
	8:00	8:05	26.6	67.0	0.2	Sunny
	10:00	10:05	33.6†	44.9†	0.2	Sunny
	12:00	12:05	29.4	54.9	0.9	Sunny
	13:52	13:57	30.1	52.5	1.0	Sunny
	15:58	16:03	28.7	53.9	0.1	Sunny
24-Oct-24	6:11	6:16	21.7	79.2	0.5	Sunny
	7:50	7:55	23.6	75.1	0.2	Sunny
	9:53	9:58	27.1	62.6	0.1	Sunny
	11:55	12:00	29.7	56.4	0.5	Sunny
	13:55	14:00	29.1	58.1	0.5	Cloudy
	15:53	15:58	27.9	62.5	0.4	Cloudy
	17:20	17:25	26.5	68.7	0.1	Sunny
25-Oct-24	6:04	6:09	23.3	87.0	0.2	Cloudy
	7:58	8:03	25.2	80.2	0.3	Sunny
	9:55	10:00	28.2	68.9	0.3	Sunny
	11:55	12:00	31.2	56.5	0.4	Sunny
	13:55	14:00	31.7	56.8	0.0	Sunny
	15:55	16:00	30.3	69.1	0.5	Sunny
	17:22	17:27	28.9	75.2	0.1	Sunny
4-Nov-24	17:20	17:25	27.2	69.9	0.0	Sunny

†Air temperature and humidity at 10:00 on 23 Oct 2024 were measured with a thermohydrometer under direct sun lights.

Table S3. Conditions of the low-temperature response test.

Test section	Start time	End time	Mean air temperature (˚C)	Mean humidity (%)
9 ˚C female	10:33 1-Nov-24	13:34 1-Nov-24	8.9	82.2
9 ˚C male	14:33 2-Nov-24	17:34 2-Nov-24	9.1	81.2
12 ˚C female	12:57 30-Oct-24	16:22 30-Oct-24	12.0	80.2
12 ˚C male	16:56 30-Oct-24	19:53 30-Oct-24	12.0	79.5
15 ˚C female	8:57 28-Oct-24	12:36 28-Oct-24	14.5	84.7
15 ˚C male	13:43 28-Oct-24	17:41 28-Oct-24	14.4	84.2
18 ˚C female	11:07 31-Oct-24	14:00 31-Oct-24	18.1	78.3
18 ˚C male	14:55 1-Nov-24	17:42 1-Nov-24	18.0	76.7
21 ˚C female	9:51 29-Oct-24	13:00 29-Oct-24	20.8†	72.7†
21 ˚C male	14:24 29-Oct-24	17:21 29-Oct-24	21.1†	73.9†
24 ˚C female	8:52 28-Oct-24	11:56 28-Oct-24	23.5†	68.9†
24 ˚C male	14:36 28-Oct-24	17:58 28-Oct-24	23.6†	69.8†

† Air temperature and humidity in the 21 and 24 ˚C sections are readings at the beginnings only, but later readings kept stable at occasional checks.

Table S4. Conditions of the long-duration flight test.

(a) The long-duration flight test

Test section	Start time	End time	Mean of air temperature (°C)	Mean of humidity (%)
10:00 start	8:45 27-May-25	10:38 28-May-25	21.0	73.8
	8:38 30-May-25	9:55 31-May-25	20.8	83.7
	8:14 5-Jun-25	9:27 6-Jun-25	21.0	80.1
18:00 start	15:56 28-May-25	17:15 29-May-25	20.7	80.2
	15:52 3-Jun-25	17:08 4-Jun-25	20.9	80.5

(b) The preliminary 14-hour flight test (males only)

Test section	Start time	End time	Mean of air temperature (°C)	Mean of humidity (%)
21℃ male	14:24 29-Oct-24	5:21 30-Oct-24	21.1†	73.9†

(a) 12L12D, the light was turned off at 18:00. (b) The males flied under light-on condition.

†Air temperature and humidity in the 21℃ male section are readings at the beginning only, but later readings kept stable at occasional checks.
